# Adrenocortical Zonation, Renewal, and Remodeling

**DOI:** 10.3389/fendo.2015.00027

**Published:** 2015-03-05

**Authors:** Marjut Pihlajoki, Julia Dörner, Rebecca S. Cochran, Markku Heikinheimo, David B. Wilson

**Affiliations:** ^1^Helsinki University Central Hospital, Children’s Hospital, University of Helsinki, Helsinki, Finland; ^2^Hochschule Mannheim – University of Applied Sciences, Mannheim, Germany; ^3^St. Louis Children’s Hospital, Washington University School of Medicine, St. Louis, MO, USA

**Keywords:** adrenal cortex, hormone, plasticity, stem cell, steroid, steroidogenesis

## Abstract

The adrenal cortex is divided into concentric zones. In humans the major cortical zones are the zona glomerulosa, zona fasciculata, and zona reticularis. The adrenal cortex is a dynamic organ in which senescent cells are replaced by newly differentiated ones. This constant renewal facilitates organ remodeling in response to physiological demand for steroids. Cortical zones can reversibly expand, contract, or alter their biochemical profiles to accommodate needs. Pools of stem/progenitor cells in the adrenal capsule, subcapsular region, and juxtamedullary region can differentiate to repopulate or expand zones. Some of these pools appear to be activated only during specific developmental windows or in response to extreme physiological demand. Senescent cells can also be replenished through direct lineage conversion; for example, cells in the zona glomerulosa can transform into cells of the zona fasciculata. Adrenocortical cell differentiation, renewal, and function are regulated by a variety of endocrine/paracrine factors including adrenocorticotropin, angiotensin II, insulin-related growth hormones, luteinizing hormone, activin, and inhibin. Additionally, zonation and regeneration of the adrenal cortex are controlled by developmental signaling pathways, such as the sonic hedgehog, delta-like homolog 1, fibroblast growth factor, and WNT/β-catenin pathways. The mechanisms involved in adrenocortical remodeling are complex and redundant so as to fulfill the offsetting goals of organ homeostasis and stress adaptation.

## Introduction

**Figure 1:**
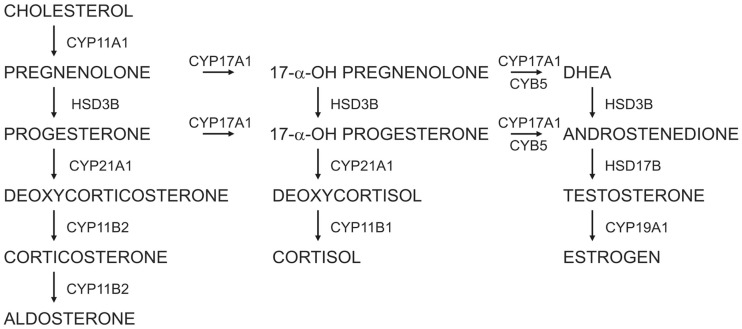
**Steroidogenic pathways in the human adrenal cortex and gonads**.

The adrenal cortex is a major source of steroid hormones, which are synthesized from cholesterol through the sequential actions of a series of cytochrome P450 (CYP) enzymes and hydroxysteroid dehydrogenases (HSDs) (Figure 1) (1). Anatomically and functionally distinct zones in the adrenal cortex synthesize specific steroid hormones in response to endocrine and paracrine signals. The regulation of adrenocortical development and homeostasis has been the subject of intensive investigation over the past decade (2–4). This review article summarizes recent advances in our understanding of adrenocortical zonation, renewal, and remodeling. Animal models useful for studies of adrenocortical biology, such as the mouse, rat, and ferret, are highlighted.

## Adrenocortical Zonation in Humans and Animal Models

**Figure 2:**
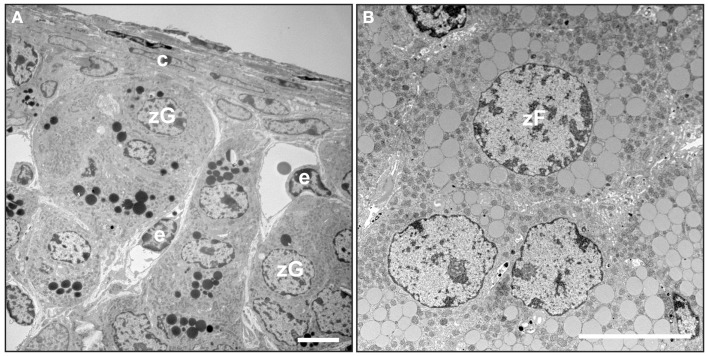
**Electron microscopy of mouse adrenal cortex**. Adrenal glands from a 4-month-old female mouse were fixed in Karnovsky’s solution, postfixed in 2% OsO_4_, dehydrated, and then embedded in epon. Thin sections were stained with uranyl acetate plus lead citrate and examined by transmission electron microscopy. **(A)** Adrenal capsule and zona glomerulosa. **(B)** Zona fasciculata. Abbreviations: c, capsule; e, endothelial cell; zF, zona fasciculata cell; zG, zona glomerulosa cell. Bars, 4 μm.

The adrenal cortex of humans is composed of three concentric layers: the zona glomerulosa (zG), zona fasciculata (zF), and zona reticularis (zR) [reviewed in Ref. (2)]. The outermost layer, the zG, functions as part of the renin-angiotensin-aldosterone system (RAAS). In response to angiotensin II (Ang II) or elevated plasma potassium ion (K^+^) concentrations, zG cells secrete aldosterone, a mineralocorticoid that induces the retention of sodium ion (Na^+^) and water and the excretion of K^+^ by the kidney. Cells in the zG express the Ang II receptor (AT1R) and aldosterone synthase (CYP11B2). At the ultrastructural level, zG cells are typified by numerous mitochondria with lamelliform cristae and a few cytoplasmic lipid droplets (Figure 2A). Cells in the zF produce glucocorticoids as part of the hypothalamic-pituitary-adrenal (HPA) axis. zF cells respond to adrenocorticotropic hormone (ACTH) via its receptor (MC2R) and the accessory protein MRAP. Cells in the zF are organized in cord-like structures, or fascicles, that are surrounded by fenestrated capillaries. Cells in this zone contain numerous mitochondria with tubulovesicular cristae, many cytoplasmic lipid droplets, and prominent smooth endoplasmic reticulum (Figure 2B) (5, 6). The innermost layer of the cortex, the zR, secretes the weak androgen dehydroepiandrosterone (DHEA) and its sulfated form DHEA-S (1). Cells of the zR resemble those of the zF but contain fewer lipid droplets and more lysosomes and vacuoles (6). The adrenal gland is covered by a fibrous capsule that serves as both a support structure and a reservoir of stem/progenitor cells for the cortex (see Section “Adrenocortical Stem Cells”) (7).

**Figure 3:**
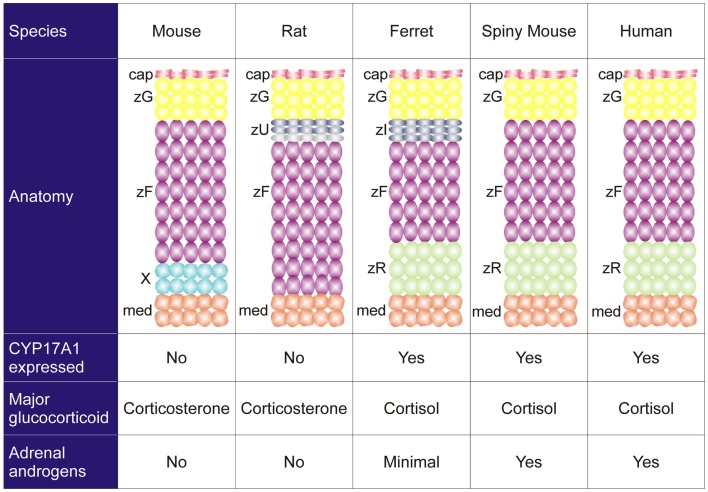
**Comparative anatomy and physiology of the adrenal cortex**. The undifferentiated zone of the rat adrenal is subdivided into outer (dark gray) and inner (light gray) zones that differ in marker expression and function (see the text). Abbreviations: cap, capsule; med, medulla; X, X-zone; zF, zona fasciculata; zG, zona glomerulosa; zI, zona intermedia; zR, zona reticularis; zU, undifferentiated zone.

Species differ in their adrenocortical zonation patterns (8) (Figure 3). In the mouse and rat, the adrenal cortex contains zG and zF, but there is no recognizable zR. The adrenal cortex of the young mouse contains an additional, ephemeral layer known as the X-zone (9, 10). The function of the X-zone remains controversial, but it may be involved in progesterone catabolism (11). The rat adrenal cortex contains a less prominent layer, the undifferentiated zone (zU), located between the zG and zF (12). The zU has been implicated in adrenocortical homeostasis and remodeling (see Section “Delta-like Homologue 1 Pathway”) (12, 13). Cells in the inner aspect of the zU express MC2R and cholesterol side-chain cleavage enzyme (CYP11A1), which catalyzes the first reaction in steroidogenesis. The inner zU lacks expression of markers of the zG (*Cyp11b2*) or zF (steroid 11β-hydroxylase; *Cyp11b1*) (14). Thus, the inner zU may represent a transitional population of cells committed to the steroidogenic phenotype. An analogous layer, the zona intermedia (zI), is present in the adrenal glands of ferrets (15). Recently, the spiny mouse (genus *Acomys*) has attracted attention as a novel model for the study of adrenocortical development and function. In contrast to the laboratory mouse (genus *Mus*), the adrenal cortex of the spiny mouse contains the zR and secretes both cortisol and DHEA (16). In this respect the adrenal gland of the spiny mouse mimics that of humans.

Species also vary in the repertoire of steroidogenic enzymes and cofactors expressed in the adrenal cortex, and these differences impact function (Figure 3). Two factors that are differentially expressed among species are 17α-hydroxylase/17,20 lyase (CYP17A1) and cytochrome b_5_ (CYB_5_). CYP17A1, a bifunctional enzyme, catalyzes the 17α-hydroxylation reaction required for cortisol synthesis and the 17,20-lyase reaction required for the androgen production (1). The lyase activity is enhanced by allosteric interactions with CYB_5_ (1). Cells in the zF and zR of humans and ferrets have 17α-hydroxylase activity, so cortisol is the principal glucocorticoid secreted by the adrenal gland of these organisms (8). In humans the adrenal cortex begins to produce DHEA and DHEA-S at adrenarche, contemporaneous with increased expression of *CYB5* in the zR (1). The adrenal glands of ferrets produce only limited amounts of androgens due to low CYB_5_ expression (8, 17). Cells in the adrenal cortex of adult mice and rats lack CYP17A1, so corticosterone is the principal glucocorticoid secreted, and adrenal androgens are not produced (8). The relative strengths and weaknesses of established and emerging animal models are summarized in Table 1.

**Table 1:** **Advantages and disadvantages of various animal models for studies of adrenocortical zonation and remodeling**.

	Mouse	Rat	Spiny mouse	Ferret
Advantages	Genetically and epigenetically tractable Well suited for transplantation experiments Gonadectomy triggers the accumulation of gonadal-like cells in the adrenal cortex (see Section “LH Signaling”)	Well suited for pharmacological studies (see Section “Adrenocortical Renewal and Remodeling”) Adrenal enucleation experiments are feasible (see Section “Adrenocortical Renewal and Remodeling”)	Adrenal gland is anatomically and functionally similar to that of humans	Well characterized neuroendocrine physiology Gonadectomy triggers the accumulation of gonadal-like cells in the adrenal cortex (see Section “LH Signaling”)
Disadvantages	Lacks zR and does not produce androgens	Lacks zR and does not produce androgens	Not widely available Not standardized with regard to genotype	Not standardized with regard to genotype

## Adrenocortical Renewal and Remodeling

The adult adrenal cortex is a dynamic tissue. Cells lost through senescence or injury are continually replenished through cell division and differentiation (2, 4). In the adult adrenal gland, most cell proliferation occurs near the periphery of the cortex, as shown by bromodeoxyuridine and [^3^H]thymidine labeling experiments [reviewed in Ref. (3)]. The remarkable regenerative capacity of the organ is evidenced by rat adrenal enucleation experiments, wherein the gland is incised and squeezed so as to extrude the cortex. Within weeks a new adrenal cortex regenerates from the remaining capsule and adherent subcapsular cells [reviewed in Ref. (18)].

Constant cellular turnover in the adrenal cortex facilitates rapid organ remodeling in response to physiological demand for steroids. Zones can reversibly enlarge, shrink, or alter their biochemical profiles to accommodate physiological needs or in response to experimental manipulations (Table 2). For example, administration of captopril, an inhibitor of the RAAS, leads to contraction of the zG in rats [reviewed in Ref. (2)].

**Table 2:** **Triggers of zonal remodeling in the adrenal cortex**.

Zone (species)	Physiological or experimental trigger	Effect	Reference
zG (rat)	↓[Na^+^] or ↑[K^+^] in diet	Expands the zone, increasing aldosterone production	(2)
	↑[Na^+^] or ↓[K^+^] in diet	Contracts the zone, decreasing aldosterone production	
zF (rat)	ACTH	Expands the zone, increasing glucocorticoid production	(2)
	Dexamethasone	Contracts the zone, decreasing glucocorticoid production	
zR (primates)	Adrenarche in humans and chimpanzees	Increases the expression of CYB_5_, enhancing DHEA production	(19)
	Social status in marmosets	Adult females develop a functional zR in a reversible manner dependent on social status	(20)
	Cortisol in human adrenocortical cells	Stimulates DHEA production through competitive inhibition of 3βHSD2 activity	(21)
X-zone (mouse)	Puberty in males or first pregnancy in females	Induces regression of the zone	(22)
	Activin	Induces regression of the zone	(23)
	Gonadectomy	Delays regression of the zone or induces growth of a secondary zone	(22, 23)

## Overview of Adrenocortical Development

**Figure 4:**
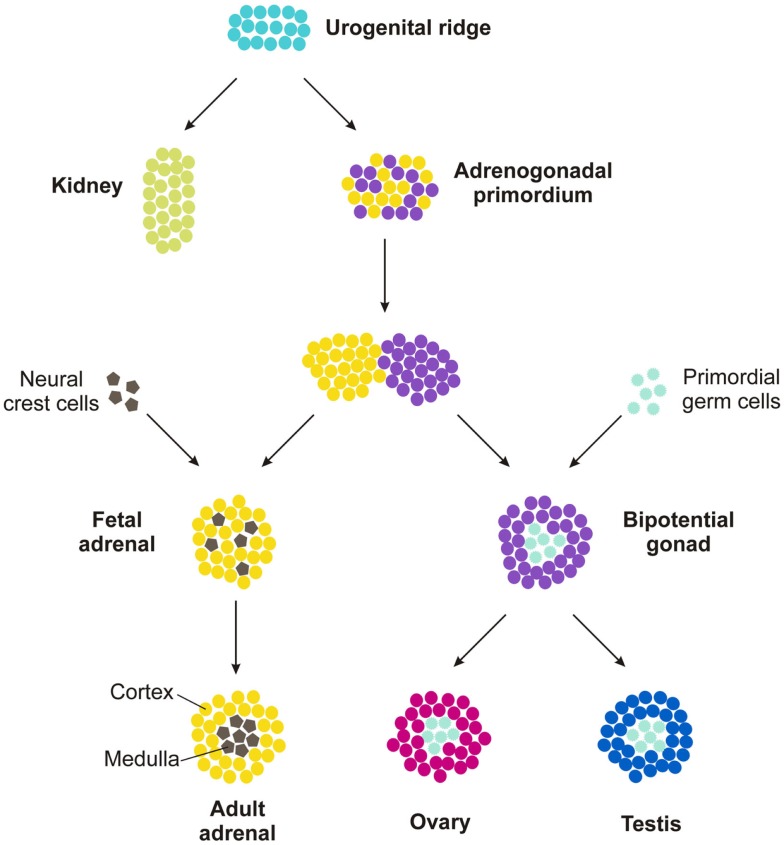
**Development of the adrenal gland and gonads**.

Embryogenesis and early postnatal development provide a contextual framework for understanding the mechanisms involved in adrenocortical zonation and homeostasis. Although structurally and functionally distinct, the adrenal cortex, ovary, and testis arise from a common progenitor, the adrenocortical primordium (AGP). The AGP is derived from a specialized region of celomic epithelium known as the urogenital ridge (Figure 4), which also gives rise to the kidney and progenitors of definitive hematopoiesis. Cells in the AGP co-express the transcription factor genes Wilms tumor suppressor-1 (*Wt1*), GATA-binding protein 4 (*Gata4*), and steroidogenic factor-1 (*Sf1*, also called *AdBP4* or *Nr5a1*) [reviewed in Ref. (2, 24, 25)]. As development proceeds, progenitors of the adrenal cortex and the gonad separate and activate different transcriptional programs. Adrenal progenitor cells in the AGP migrate dorsomedially into subjacent mesenchyme, upregulate expression of *Sf1*, and downregulate expression of *Wt1* and *Gata4* (25, 26). In contrast, gonadal progenitor cells in the AGP migrate dorsolaterally and maintain expression of *Sf1*, *Wt1*, and *Gata4*. Adrenal precursors combine with neural-crest derived sympathoblasts, the precursors of chromaffin cells in the medulla, to form the adrenal anlagen. Gonadal progenitors combine with primordial germ cells to form the bipotential gonad. Subsequently, the nascent adrenal glands become enveloped by capsule cells, which are derived from both surrounding mesenchyme and fetal adrenal cells that previously expressed *Sf1* [reviewed in Ref. (27)].

In rodents, zonal patterns of steroidogenic enzyme expression first become evident during embryonic development [reviewed in Ref. (24)]. In mice, expression of *Cyp11a1* is first detectable in the nascent adrenal at embryonic day (E) 11.5–12.5 (26, 28), and there is a concurrent increase in the level of endogenous biotin (29). Expression of the zF marker *Cyp11b1* begins at E13.5, whereas expression of the zG markers Ang II receptor type 1 (*At1b*) and *Cyp11b2* appears in the periphery of the cortex just before birth, and *Cyp11b2* and *Cyp11b1* expression domains are mutually exclusive at this stage (30–32).

By the eighth week of gestation in humans, the fetal adrenal cortex contains two morphologically distinct layers: an inner fetal zone (Fz) and an outer definitive zone (Dz) (33). The Fz is thick and contains large, eosinophilic cells, whereas the Dz is thin and contains small, basophilic cells. Functionally, the Fz resembles the adult zR. The Fz expresses *CYP17A1* and *CYB5* and produces large amounts of DHEA and DHEA-S, which are converted by the sequential actions of the liver and placenta into estrogens. A third cortical zone, termed the transitional zone (Tz), becomes evident shortly thereafter. The Tz produces cortisol, and an early burst of cortisol production during the ninth week of gestation, coinciding with a transient increase in expression of 3β-hydroxysteroid dehydrogenase type 2 (*HSD3B2*), is thought to safeguard female sexual development by suppressing the fetal HPA axis and thereby inhibiting adrenal androgen production (34). At birth, the adrenal gland is almost as large as the kidney, but the size of the organ decreases dramatically over first 2 weeks of neonatal life; the Fz involutes via apoptosis, and there is a concomitant reduction in adrenal androgen production (1). The mouse X-zone, a remnant of the fetal adrenal that regresses postnatally (9), is thought to be the analog of the human Fz. Postnatally, the human Dz differentiates into the anatomically and functionally distinct zones of the adult cortex.

## Adrenocortical Stem Cells

The adrenal cortex contains stem/progenitor cells that can divide and differentiate to replenish senescing cells and maintain or expand zones (Table 3) [reviewed in Ref. (4)]. In one long-standing model of adrenal zonation, the cell migration model, stem/progenitor cells in the periphery of the adrenal cortex differentiate and migrate centripetally to repopulate the gland before undergoing apoptosis in the juxtamedullary region (35). Aspects of this model have been validated through lineage tracing analyses (24, 30, 36), but recent studies indicate that the regulation of zonation is more complex than originally appreciated [reviewed in Ref. (13)]. It is now clear that distinct pools of stem/progenitor cells exist in the adrenal capsule, subjacent cortex, juxtamedullary region, and other sites (Table 3). Some of these pools appear to be activated only during specific developmental windows or in response to extreme physiological demand. Under certain experimental conditions, adrenocortical zones can be replenished by centrifugal migration (37, 38). For example, stem/progenitor cells in the juxtamedullary region can proliferate, differentiate, and centrifugally repopulate the cortex with fetal-like cells, as is seen in gonadectomy (GDX)-induced secondary X-zone formation and in a genetic model of dysregulated cAMP production (37, 39, 40). The mechanisms that govern centripetal and centrifugal migration are not well understood. Whether centrifugal migration operates under basal conditions is unknown.

**Table 3:** **Stem/progenitor cell populations that give rise to steroidogenic and non-steroidogenic cells in the adrenal cortex**.

Stem/progenitor population	Location	Comments	Reference
WT1^+^ progenitors	Capsule	Under basal conditions, WT1^+^ capsule cells give rise to steroidogenic cells in the adrenal cortex. GDX triggers their differentiation into gonadal-like tissue	(25)
GLI1^+^ progenitors	Capsule	In response to SHH, GLI1^+^ progenitors migrate into the cortex and differentiate into steroidogenic cells	(27, 30, 41)
TCF21^+^ progenitors	Capsule	TCF21^+^ capsular cells give rise to non-steroidogenic stromal cells in the adrenal cortex	(27)
SHH^+^ progenitors	Subcapsular region	These progenitors give rise to steroidogenic cells in the zF and zG but not capsule cells	(27, 30, 41)
Fetal adrenal-like progenitors	Juxtamedullary region	These progenitors, normally dormant in the adult, can become activated following certain experimental manipulations and migrate centrifugally	(37, 39, 40)

*These progenitor populations, defined by fate mapping studies and related techniques, are not mutually exclusive. For example, WT1^+^ progenitors have been shown to co-express *Gli1* and *Tcf21*. Some of these progenitors give rise to differentiated cells only during specific developmental windows or in response to experimental manipulation*.

## Adrenocortical Cell Plasticity

Cell plasticity is another mechanism for replenishing adrenocortical cells lost to senescence or injury. Plasticity refers to the ability of cells to adopt an alternate functional identity in response to cues from the hormonal milieu and cellular microenvironment. One form of plasticity entails trans-differentiation, the direct conversion of one differentiated cell into a differentiated cell of another lineage (42). A second form of plasticity involves de-differentiation, wherein a differentiated cell reverts to a less differentiated cell within the same tissue lineage (42). Interconversion of differentiated cells, either through trans- or de-differentiation, provides an alternative to regeneration via mobilization of stem/progenitor cells. Such functional redundancy ensures organ homeostasis and an optimal adaptation to stress (13).

**Figure 5:**
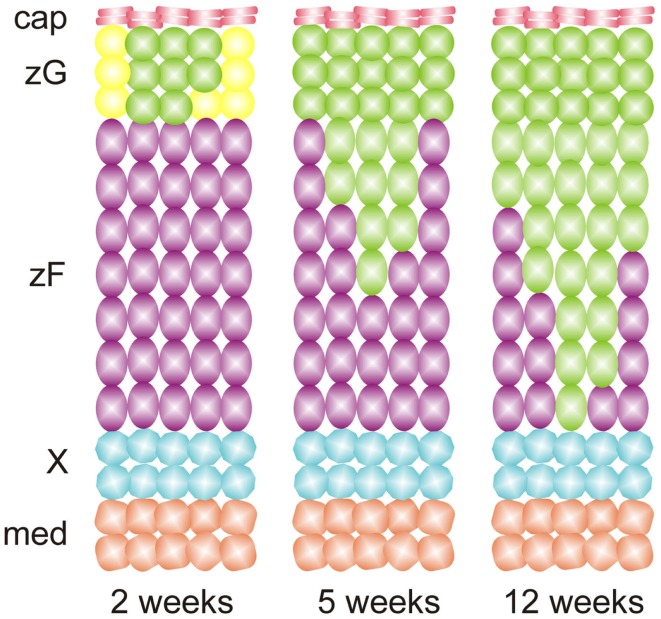
**Adrenocortical zonation during postnatal mouse development results from lineage conversion of zG cells into zF cells, as evidenced by fate mapping using *Cyp11b2*-cre and a GFP reporter**. Recombination of the reporter in zG leads to expression of GFP (green cells). The resultant cells migrate inward and differentiate into zF cells. Abbreviations: cap, capsule; med, medulla; X, X-zone; zF, zona fasciculata; zG, zona glomerulosa.

The plasticity of differentiated adrenocortical cells was elegantly demonstrated in fate mapping studies by Freedman et al. (36), who used *Cyp11b2-*Cre to permanently mark zG cells and their descendants with green fluorescent protein (GFP). By tracing the fate of GFP^+^ cells, the investigators showed that adrenocortical zonation is orchestrated in part by direct lineage conversion of zG cells into zF cells (Figure 5). To show that zG-to-zF conversion participates in adrenocortical remodeling, Freedman et al. treated adult mice with glucocorticoids to inhibit the HPA axis (36). Glucocorticoid treatment caused contraction of the zF and loss of GFP^+^ cells in this zone. Following withdrawal of exogenous glucocorticoids, zG-to-zF conversion resumed and the zF expanded. Remarkably, when conversion of zG to zF cells was abrogated through conditional deletion of the *Sf1* gene in CYP11B2^+^ cells, a functional zF still formed, implying the existence of alternate routes for differentiation of zF cells. These alternative sources for zF cells remain the subject of active investigation. Collectively, these results support a model in which differentiated cells undergo lineage conversion during adrenocortical renewal and remodeling.

## Developmental Signaling Pathways Implicated in Adrenocortical Zonation, Renewal, or Remodeling

Developmental signaling pathways control cell pluripotency, differentiation, and patterning in various tissues. As detailed below, some of these signaling pathways play key roles during the exponential growth phase of adrenal cortex development (12, 24, 43, 44). Additionally, these pathways regulate renewal and remodeling in the adult organism.

### Hedgehog pathway

The hedgehog family of morphogens comprises sonic hedgehog (SHH), Indian hedgehog, and desert hedgehog. Each of these ligands binds to Patched-1 (PTCH1), a transmembrane receptor that is expressed on target cells (45). In the absence of hedgehog binding, PTCH1 inhibits the G protein-coupled receptor Smoothened (SMO) [reviewed in Ref. (2, 46)]. As a result, the zinc finger transcription factors GLI2 and GLI3 are proteolytically digested and lose their activation domains (47). The resultant truncated forms of GLI2 and GLI3 repress transcription. Binding of hedgehog ligands to PTCH1 relieves the inhibition it exerts on SMO, thereby preventing the proteolytic processing of the GLI factors. Full-length GLI2 and GLI3 act as transcriptional activators. The related transcriptional activator, GLI1, is not expressed in the absence of hedgehog ligand, but is upregulated by activation of the pathway. Consequently *Gli1* expression serves as a useful marker for active hedgehog signaling (48).

**Figure 6:**
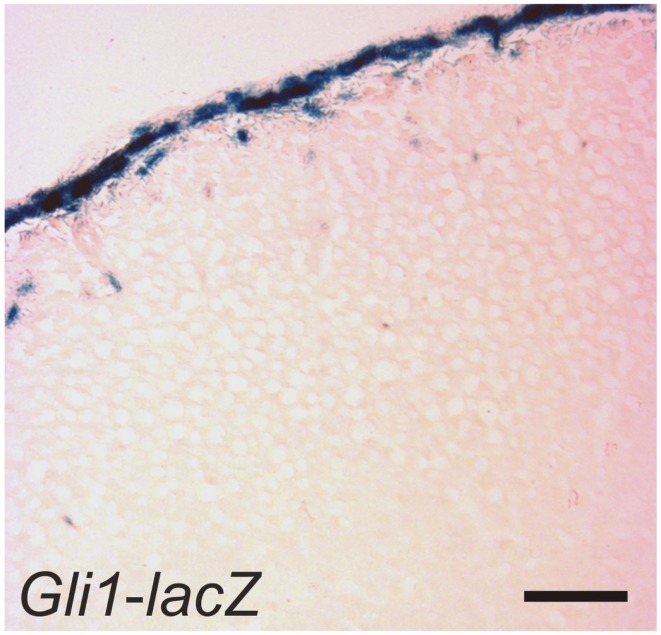
**GLI1^+^ cells in the adrenal capsule**. An adrenal gland from a 1-month-old female *Gli1-lacZ* mouse was whole mount stained with X-gal, cryosectioned, and counterstained with eosin. Bar, 50 μm.

SHH, the only member of the hedgehog family produced in the adrenal cortex, is secreted by subcapsular cells that express *Sf1* but not the terminal enzymes required for corticoid synthesis (30, 41, 49). Capsular cells, which do not express *Sf1*, respond to SHH by expressing *Gli1* (Figure 6). Some of these GLI1^+^ capsule cells migrate centripetally into the cortex, lose responsiveness to SHH, and become steroidogenic, as evidenced by upregulation of *Sf1* and differentiation markers characteristic of the zG (*Cyp11b2*) or zF (*Cyp11b1*) (Table 2). GLI1^+^ progenitor cells efficiently contribute to steroidogenic lineages during the exponential phase of cortical growth in embryo, fetus, and newborn mouse (30). In the adult mouse, GLI1^+^ progenitors contribute to the cortex with low efficiency, but the pathway can be activated in the adult following experimental manipulations such as dexamethasone-induced cortical atrophy. Conditional deletion of *Shh* in steroidogenic cells of the mouse adrenal results in cortical hypoplasia and capsular thinning, but does not cause major alterations in zonation (30, 41, 49).

### Delta-like homolog 1 pathway

A related signaling protein implicated in adrenocortical homeostasis is Delta-like homolog 1 (DLK1). This factor, also known as preadipocyte factor-1 (PREF-1), is a transmembrane protein related to the Notch family of signaling molecules. DLK1 was originally identified as an important regulator of the undifferentiated state in preadipocytes (50). Cleavage of the extracellular domain of DLK1 by TNF-α converting enzyme produces a biologically active soluble peptide that inhibits the differentiation of preadipocytes into mature adipocytes (50). Subsequent studies showed that DLK1 controls the quiescence of stem/progenitor cells in not only adipose tissue but also other tissue types, including the adrenal cortex (12, 50).

**Figure 7:**
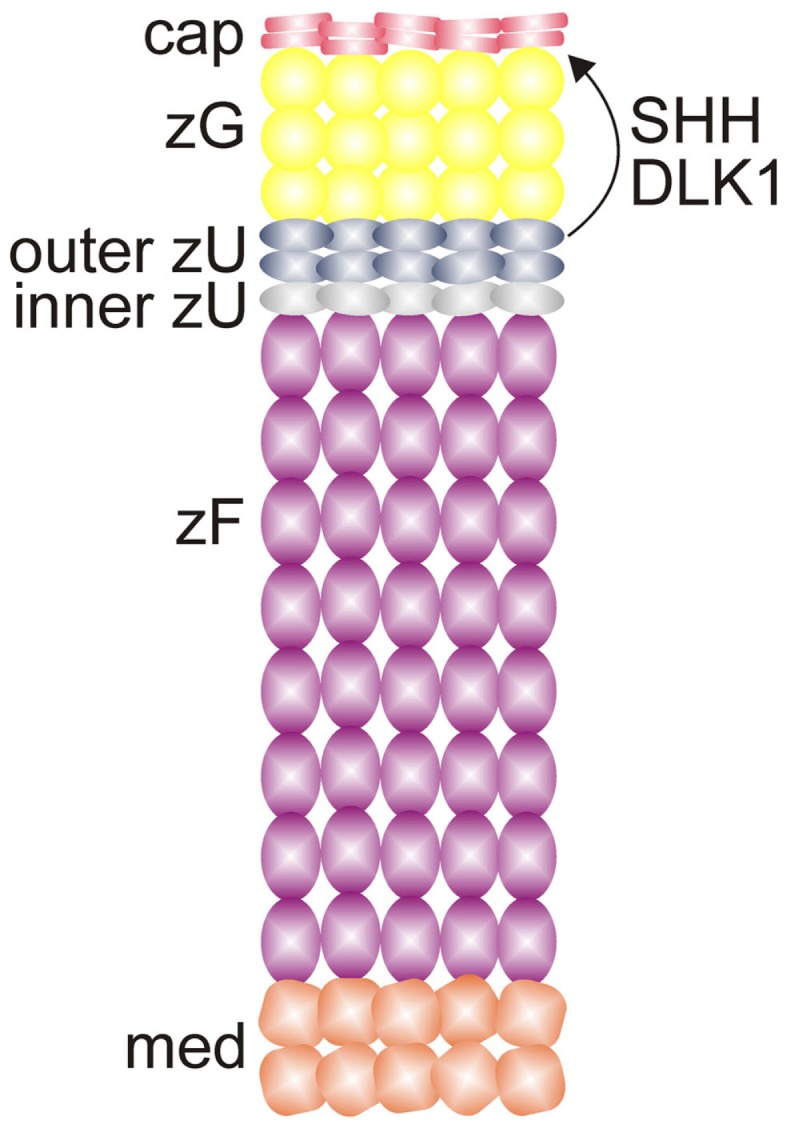
***SHH* and *DLK1* are co-expressed in the outer zU of the rat adrenal cortex and may act in concert to regulate stem/progenitor cells in the adrenal capsule**. Abbreviations: cap, capsule; DLK1, delta-like homolog-1; med, medulla; SHH, sonic hedgehog; X, X-zone; zF, zona fasciculata; zG, zona glomerulosa; zU, undifferentiated zone.

Adrenal enucleation experiments have shown that *Dlk1* expression is downregulated and not re-established until zonation of the cortex is complete, suggesting that DLK1 is a negative regulator of adrenocortical differentiation (51). *Dlk1* is co-expressed with *Shh* in the outer zU of the rat (Figure 7) (12). Soluble DLK1, like SHH, modulates *Gli1* expression in nearby capsule cells. In addition to being co-expressed, *Dlk1* and *Shh* are coordinately regulated (12). Both genes are downregulated in the adrenals of mice fed a low Na^+^ diet. Conversely, *Dlk1* and *Shh* are upregulated in the adrenals of mice treated with captopril. These findings suggest that DLK1 and SHH may act together to fine tune the activation of signal receiving cells in the adrenal capsule of the rat. The expression pattern of *Dlk1* differs between rats and mice; in mice *Dlk1* is expressed in the adrenal capsule rather than the underlying cortex. Nevertheless, indirect evidence suggests that in mice, as in rats, DLK1 may negatively regulate the differentiation of GLI1^+^ capsular progenitor cells (43).

### Fibroblast growth factor pathway

Mouse genetic studies have implicated the FGF signaling pathway in adrenocortical development and maintenance [reviewed in Ref. (2, 43)]. The FGF family comprises a large group of extracellular ligands that signal through a family of tyrosine kinase receptors, the FGF receptors (FGFRs). In mammals, the FGFR family consists of four genes, FGFR1-4, which undergo alternative splicing to generate an array of receptors that differ in ligand affinities (52). In the presence of heparin, FGFs bind to their cognate receptors, promoting receptor dimerization and autophosphorylation. This in turn stimulates downstream signaling pathways, including the phosphatidylinositol 3-kinase (PI3K), Janus kinase and signal transducer and activator of transcription (JAK–STAT), and mitogen-activated protein kinase (MAPK) pathways. FGF signaling is essential for proper patterning of the embryo, and this pathway participates in stem cell maintenance (53). Factors in the FGF pathway are expressed in both the adrenal capsule and cortex, as summarized in Table 4.

**Table 4:** **FGF ligands and receptors implicated in adrenocortical cell development and homeostasis**.

	Protein	Location	Comments	Reference
Ligands	FGF1	Cortex	This isoform activates FGFR2 IIIb	(43)
	FGF2	Capsule	FGF2, which activates FGFR1 IIIc, acts as a mitogen for adrenocortical cells both in culture and in gland regeneration experiments and has been shown to bind specifically to cells from the zG	(43, 54–58)
	FGF9	Capsule	This isoform activates FGFR1 IIIc	(43)
Receptors	FGFR1 IIIc	Capsule and cortex	This FGFR isoform is expressed in both capsule and cortex, although its precise role in adrenocortical development is unknown	(43)
	FGFR2 IIIb	Cortex	Like SHH and β-catenin, this FGFR isoform is expressed in the subcapsular region; embryos with a global *Fgfr2 IIIb* deletion have hypoplastic adrenal glands, impaired steroidogenesis, and thickened adrenal capsules with increased *Gli1* expression	(43, 59)
	FGFR2 IIIc	Cortex	Like SHH and β-catenin, this FGFR isoform is expressed in clusters of cells in the subcapsular region. Deletion of both FGFR2 isoforms in steroidogenic tissues leads to hypoplastic adrenals	(43, 60)
	FGFR3 IIIc	Cortex	This isoform is expressed in cortex, although its precise role in adrenocortical development is unknown	(43)

### WNT/β-catenin signaling

**Figure 8:**
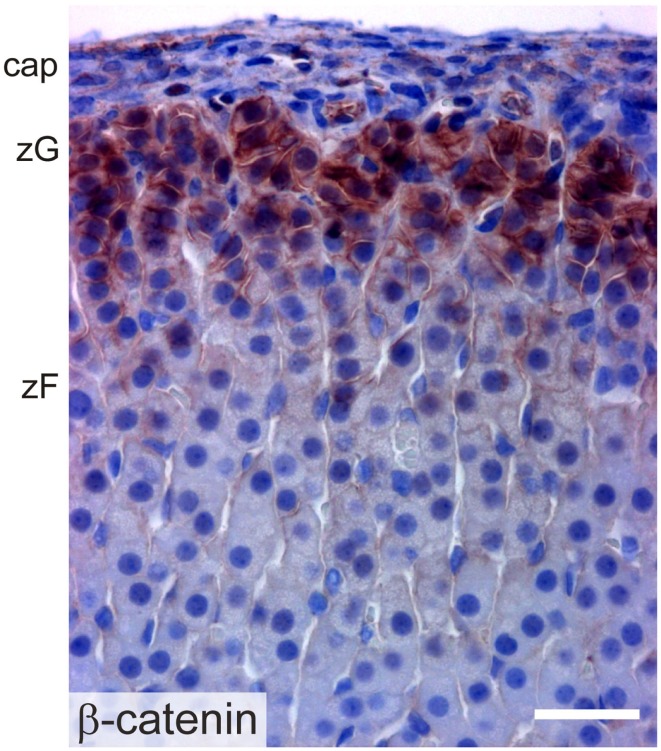
**Immunoperoxidase staining of β-catenin in the adrenal cortex of a 2-month-old female mouse**. Abbreviations: cap, capsule; zF, zona fasciculata; zG, zona glomerulosa; Bar, 30 μm.

β-catenin exists in two pools: a cytoskeletal pool controls the interaction of cadherin complexes with adherens junctions, while a cytoplasmic pool participates in canonical WNT signaling, acting as a co-activator for transcription factors of the TCF/LEF family [reviewed in Ref. (2)]. Transcriptionally active β-catenin has been demonstrated in the AGP, the adrenal primordium, and adrenal subcapsular cells of the fetus and adult (61) (Figure 8). WNT/β-catenin signaling is thought to maintain the undifferentiated state of adrenocortical stem/progenitor cells (7, 62). Targeted mutagenesis of β-catenin in SF1^+^ cells causes late onset adrenal hypoplasia, presumed to be the result of stem/progenitor cell pool depletion (61). On the other hand, constitutive activation of β-catenin in steroidogenic cells expressing aldo-keto reductase family 1, member B7 (*Akr1b7*) causes abnormal accumulation of undifferentiated cells in the capsule and subcapsule and a concomitant increase in *Shh* mRNA expression (40).

Regulation of the WNT/β-catenin pathway is complex and entails not only a family of WNT ligands but also multiple receptors, co-receptors, decoy receptors, and other modulators (Table 5). This complexity allows fine tuning of the response to morphogen gradients. Stem cell self-renewal mechanisms are frequently co-opted to drive oncogenesis, and WNT signaling is the pathway most frequently mutated in adrenocortical carcinomas (63) (Table 5).

**Table 5:** **Factors implicated in WNT/β-catenin signaling in the adrenal cortex**.

Factor	Function	Adrenocortical phenotypes	Reference
WNT4	Ligand that activates signaling	*Wnt4^-/-^* mice have impaired zG differentiation and decreased aldosterone production	(69)
Frizzled (FZD)	Receptor for WNTs		(70)
LDL receptor-related proteins 5 and 6 (LRP5/6)	Co-receptors for WNTs		(44)
R-spondin-3 (RSPO3)	Ligand that potentiates WNT signaling		(71)
Leucine-rich repeat containing G protein-coupled receptor 5 (LGR5)	Receptor for RSPO3; inhibits the activity of ZNRF3		(72)
Zinc and ring finger 3 (ZNRF3)	E3 ubiquitin ligase that inhibits signaling by promoting the degradation of FZD/LRP	Somatic mutations in *ZNRF3* are common in human adrenocortical carcinomas	(73)
Secreted frizzled related proteins (SFRP1/2)	Decoy receptors that inhibit signaling by sequestering WNT ligands away from activating receptors	The *Sfrp1* locus has been linked to GDX-induced adrenocortical neoplasia in the mouse; decreased expression of *SFRP2* is associated with aldosterone-producing adenoma development	(66, 74)
Dickkopf-3 (DKK3)	Inhibits signaling by interacting with LRPs	*Dkk3* expression is greater in the zG than in other zones. Genetic studies indicate that *Dkk3* regulates aldosterone biosynthesis	(70, 75)
Kringle containing transmembrane protein 1 (KREMEN1)	Inhibits signaling by binding DKK3 and LRPs and inducing internalization of FZD	Somatic mutations in *KREMEN1* are common in human adrenocortical carcinomas	(63)

In addition to its proposed role in stem cell maintenance and recruitment, the WNT/β-catenin pathway has been implicated in tissue patterning in the adult organism. For example, proper zonation of the liver requires restriction of WNT/β-catenin signaling to hepatocytes near the central vein (64). In an analogous fashion, restriction of WNT signaling to the periphery of the adrenal cortex is thought to direct zonation in this tissue. Constitutive activation of β-catenin signaling in the mouse zF using *Akr1b7-*cre triggers the ectopic expression of the zG marker *Cyp11b2* and increased production of aldosterone (40, 65). Moreover, studies have shown that β-catenin directly regulates the expression of genes critical for zG function, including *At1r* and *Cyp11b2* (66).

Recent studies have shown that proper differentiation of zF cells requires suppression of WNT/β-catenin signaling (67). *In vitro* treatment of a zF cell line (ATCL7) with a chemical inducer of canonical WNT signaling (BIO) resulted in down-regulation of genes essential for zF function, including *Mc2r*, *Cyp11a1*, and *Cyp11b1* (68). Promoter analyses suggested that the molecular basis for this repression may involve the displacement of SF1 from steroidogenic gene promoters by β-catenin (68). These experiments also identified CCDC80 as a novel secreted inhibitor of zF steroidogenesis. Collectively these studies suggest that coordinated regulation of WNT/β-catenin signaling is critical for adrenocortical patterning; WNT/β-catenin signaling must be active for zG determination and must be extinguished for zF determination.

## Other Signaling Pathways Implicated in Adrenocortical Growth and Remodeling

Adrenocortical growth and homeostasis are controlled by a diverse array of endocrine/paracrine factors, including ACTH, Ang II, and insulin-related growth factors (IGFs) (15, 24). Hormones traditionally associated with reproductive function, including luteinizing hormone (LH), activin, inhibin, and prolactin, also influence the differentiation and function of adrenocortical cells [reviewed in Ref. (15)].

### cAMP signaling

Many of the hormones that regulate adrenocortical cell proliferation bind to G-protein coupled receptors on the surface of cells [reviewed in Ref. (38)]. Activation of these receptors stimulates adenylate cyclase, resulting in cAMP production. cAMP binds to the regulatory subunits of PKA, allowing the catalytic subunits of protein kinase A (PKA) to phosphorylate downstream effectors, including transcription factors that enhance expression of steroidogenic genes (38).

Inactivating mutations in the protein kinase-A regulatory subunit gene (*PRKAR1A*) lead to excessive cAMP production. Such mutations cause Carney complex, a syndrome associated with pituitary-independent Cushing syndrome and adrenocortical neoplasia. Conditional deletion of *Prkar1a* in the adrenal cortex of mice (using *Akr1b7-*cre) leads to disrupted stem/progenitor cell differentiation, excess cell proliferation, and impaired apoptosis in the adrenal cortex (37). This resistance to apoptosis is mediated in part by crosstalk between the PKA and mammalian target of rapamycin (mTOR) pathways (39). As these mice age, a new zone composed of cells that express *Cyp17a1* and secrete cortisol appears in the inner aspect of the cortex. This ectopic X-like zone is thought to arise from normally dormant stem/progenitor cells in the juxtamedullary region (37, 38). These studies and others (38) indicate that normal adrenocortical cell differentiation and proliferation require proper regulation of PKA activity.

### IGF signaling

This pathway has been implicated in growth and differentiation of adrenocortical cells. The IGF family consists of two ligands, IGF1 and IGF2, which bind to the receptor tyrosine kinase IGF1R and promote mitosis/survival via signaling through the MAPK and PI3K pathways (76, 77). *IGF1* and *IGF2* are expressed at comparable levels in the adult adrenal cortex, whereas *IGF2* is highly and preferentially expressed in the fetal adrenal cortex. IGF1R is enriched in the subcapsular region (78). The activity of IGFs is modulated by a family of six IGF-binding proteins (IGFBPs), which can bind and either stimulate or inhibit the activity of IGFs (76).

Mice deficient in both the *Igf1r* and the insulin receptor (*Insr*) genes exhibit adrenal agenesis and male-to-female sex reversal (79). The AGP of the double knockout mice contains half the number of SF1^+^ cells found in wild-type mice. These data indicate that IGF signaling is pivotal for adrenocortical cell specification. Additionally, IGFs have been shown to enhance basal and ACTH-induced steroidogenesis in fetal and adult adrenocortical cells (80).

### Transforming growth factor β signaling

The Transforming growth factor β (TGF-β) signaling pathway has been implicated in the maintenance and differentiation of stem/progenitor cells (81). The TGF-β superfamily consists of a diverse array of ligands. Two members of this family, activin and inhibin, are expressed in the fetal and adult adrenal cortex, and have been shown to regulate the growth, function, and survival of adrenocortical cells. Activin signaling is mediated by type I and type II receptors, which are integral membrane receptor serine/threonine kinases. Intracellular SMAD proteins transduce signals from these receptors to the nucleus (81). Activin has been shown to inhibit adrenocortical cell growth, enhance apoptosis of X-zone cells, and modulate steroidogenesis (23, 82, 83). By binding beta-glycan and ActRIA, inhibin blocks activin binding to the type II receptor and subsequent recruitment of the signaling type I receptor (83).

Following GDX, ovarian-like tissue accumulates in the adrenal cortex of *Inha*^-/-^ mice in an LH dependent manner (23, 84, 85). The loss of *Inha* results in constitutive TGF-β2 activation in adrenocortical progenitor cells, with subsequent expansion of cells that express *Gata4* and other gonadal-like markers. Thus, *Inha* impacts cell fate decisions (adrenal *vs*. gonadal) in adrenal cortex.

### LH signaling

This glycoprotein hormone is composed of a common gonadotropin α-subunit and hormone-specific β-subunit. LH is secreted from the pituitary in response to gonadotropin releasing hormone (GnRH). LH binds to G-protein–coupled surface receptor, LHCGR, present on gonadal steroidogenic cells and activates downstream signals, including the cAMP/PKA, MAPK, and PI3K pathways (15). This in turn leads to enhanced expression of steroidogenic enzyme genes, resulting in increased production of sex steroids. Activation of LHCGR also has pleiotropic effects on cell growth and differentiation.

Cells in the adrenal glands express LHCGR and can respond to surges in LH, as evidenced by the phenomenon of GDX-induced adrenocortical neoplasia (71). Following GDX, gonadal-like neoplasms accumulate in the subcapsular region of the adrenal cortex of certain strains of mice. This phenomenon is thought to reflect LH-induced metaplasia of stem/progenitor cells in the adrenal cortex, although the term “neoplasia” is used more often than “metaplasia” to describe the process, because with time these lesions can evolve into frank adenomas or carcinomas. The neoplastic cells express gonadal-like markers (e.g., *Lhcgr*, *Gata4*, and *Cyp17a1*) and secrete sex steroids (86). This phenomenon occurs in other species such as ferrets and goats [reviewed in Ref. (71)]. Moreover, adrenocortical tumors with histologic features resembling luteinized ovarian stroma (“thecal metaplasia”) have been reported, albeit rarely, in postmenopausal women and men with acquired testicular atrophy. Genetic and pharmacologic experiments using mice or ferrets support the premise that LH has a central role in GDX-induced adrenocortical neoplasia [reviewed in Ref. (15, 71)]. The formation of ectopic gonadal-like tissue in the adrenal gland can be viewed as an extreme example of adrenocortical remodeling in response to GDX (13, 25).

## Transcription Factors Implicated in Renewal and Remodeling

### SF1

SF1 is a master regulator of adrenocortical development and the prototype of steroidogenic transcription factors. SF1 regulates a wide array of genes required for steroidogenic cell function (87, 88). Traditionally, SF1 has been classified as an orphan nuclear receptor, but recent studies have shown that certain phospholipids and sphingolipids bind and regulate this transcription factor [reviewed in Ref. (89)]. For example, the activity of SF1 can be modulated by phosphorylation of the 3-position of the inositol head group of phosphatidylinositol-4,5-bisphosphate PI(4,5)P_2_ while this phospholipid is bound to SF1 (90). Thus, it is hypothesized that multiple bioactive lipids function as ligands for SF1 and differentially regulate SF1 activity in a context-dependent manner (89).

*Sf1*^-/-^ mice exhibit degeneration of the AGP due to apoptosis, which results in agenesis of both the adrenal glands and gonads (91). Similarly, targeted mutagenesis of transcription factors that activate *Sf1* expression, such as *Wt1*, *Pbx1*, and *Cited*, severely impairs adrenal gland development [reviewed in Ref. (25, 26, 92)]. *Sf1*^±^ mice have small adrenal glands, reduced corticosterone production in response to stress, and impaired compensatory growth response following unilateral adrenalectomy (91, 93). Individuals with mutations in the DNA-binding domain of SF1 exhibit primary adrenal failure and gonadal dysgenesis. In addition to regulating steroidogenesis, this transcription factor has been implicated in the control of other fundamental cellular processes including glycolysis (87, 88).

Mice harboring multiple copies of *Sf1*, mimicking the amplification of *Sf1* seen in childhood adrenocortical carcinoma (94, 95), develop adrenocortical neoplasms that express gonadal-like markers. This suggests that SF1 can influence cell fate determination. Intriguingly, genetic ablation of the SF1 target gene *Vnn1*, encoding the gonadal-like marker Vanin-1, has been shown to reduce the severity of neoplastic lesions in the *Sf1* transgenic mice (96). Similarly, mice in which the endogenous *Sf1* gene of the mouse has been replaced with a mutant lacking a key SUMOylation site exhibit abnormal cell fate specification in steroidogenic tissues, including ectopic expression of gonadal markers (97). The mutant mice also exhibit persistence of the X-zone (97).

### Dosage-sensitive sex reversal, adrenal hypoplasia critical region on chromosome X (DAX1)

The activity of SF1 is modulated by *Dax1* (also called *Nr0b1*), an X-linked gene that encodes a repressor of steroidogenic gene expression (98). In response to ACTH, SF1-positive subcapsular progenitors downregulate *Dax1* and differentiate into adrenocorticoid-producing cells. DAX1 deficiency in humans and mice leads to excessive differentiation of subcapsular progenitors and eventual depletion of the stem/progenitor cell compartment (99, 100). Cytomegaly, a hallmark of adrenal dysfunction associated with *Dax1* deficiency (98, 99, 101), is thought to be a compensatory response to a reduced number of cortical cells or to progenitor cell exhaustion (100).

### TCF21

TCF21 (also known as POD1) is a basic helix-loop-helix transcription factor functions as a repressor of *Sf1* (102). *Tcf21* is expressed in the adrenal capsule of adult mice (103), and adrenal glands from *Tcf21^-/-^* mice exhibit ectopic expression of *Sf1* in the capsule (103). As mentioned previously, some capsule cells are derived from progenitors in the fetal adrenal cortex, and it has been proposed that TCF21 downregulates *Sf1* expression in these cells upon recruitment into the capsule (27). Lineage tracing studies have shown that TCF21^+^ capsular cells give rise to non-steroidogenic stromal cells in the adrenal cortex, but not to steroidogenic cells (27). Collectively these studies suggest that TCF21^+^ cells in the adrenal capsule participate in adrenocortical homeostasis.

### WT1

**Figure 9:**
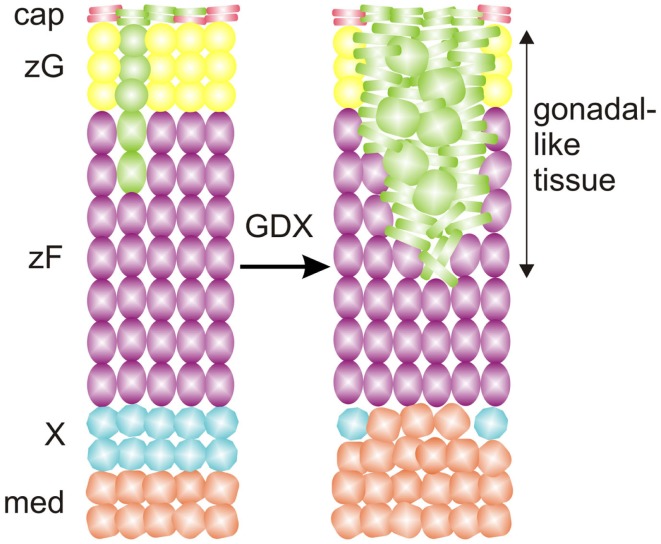
**WT1 marks a population of AGP-like progenitors within the adrenal capsule of the mouse**. Under basal conditons, AGP-like cells give rise to normal steroidogenic cells in the cortex, as evidenced by lineage tracing analysis with a GFP reporter. Gonadectomy (GDX) triggers the differentiation of AGP-like cells into wedges of gonadal-like steroidogenic tissue. Secretion of sex steroids and other hormones by the ectopic gonadal tissue causes regression of the subjacent X-zone. Abbreviations: cap, capsule; med, medulla; X, X-zone; zF, zona fasciculata; zG, zona glomerulosa.

Fate mapping studies of WT1^+^ cells have identified long-lived progenitor population in the adrenal capsule characterized by expression of *Wt1* and *Gata4*, markers of the AGP (25, 104). Under basal conditions these AGP-like cells give rise to normal adrenocortical cells (Figure 9). GDX activates these WT1^+^ progenitors and drives their differentiation into gonadal-like steroidogenic tissue. Hence, WT1^+^ capsular cells represent a reserve stem/progenitor cell population with AGP-like features that can be mobilized in response to extreme physiological demand (i.e., the hormonal changes associated with GDX).

In the mouse embryo *Wt1* repression is necessary for proper expression of *Sf1* and differentiation of stem/progenitor cells into adrenocortical cells (25, 104). Ectopic expression of a transcriptionally active isoform of WT1 in SF1^+^ progenitors causes adrenocortical hypoplasia, increased expression of *Gata4*, *Gli1*, and *Tcf21*, and contraction of the X-zone. WT1 directly regulates the expression of *Gli1* in adrenal tissue suggesting that ectopic expression of *Wt1* prevents differentiation into SF1^+^ adrenocortical steroidogenic cells by maintaining cells in a GLI1^+^ progenitor state.

### GATA binding protein-6 (GATA6)

This transcription factor is expressed in the adrenal cortex of the fetal mouse (105). Postnatally, adrenal expression of *Gata6* is limited to capsular and subcapsular cells (106). Targeted deletion of *Gata6* in SF1^+^ cells results in a pleiotropic adrenal phenotype that includes a thin adrenal cortex, cytomegaly, blunted corticoid production, ectopic chromaffin cells, and aberrant expression of gonadal-like markers (106). Thus, GATA6 is thought to limit the differentiation of adrenal stem/progenitor cells into gonadal-like cells.

**Figure 10:**
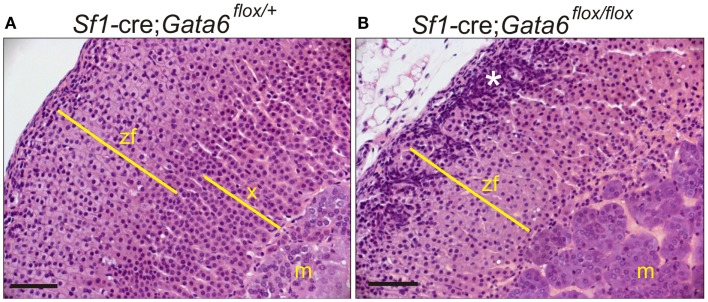
**GATA6 is required for formation of a secondary X-zone**. **(A,B)** 3-week-old *Sf1-cre; Gata6^flox/^*^+^ or *Sf1-cre; Gata6^flox/flox^* mice were orchiectomized. Adrenal tissue was harvested 1 month later, and paraffin sections were stained with H&E. Note the absence of a secondary X-zone in the mutant mice. The asterisk highlights gonadal-like cells in the subcapsular region. Bar, 50 μm.

*Gata6* mutant mice also exhibit abnormal adrenocortical zonation: virgin females lack an X-zone, and castrate males lack a secondary X-zone (Figures 10A,B) (106). *Gata6* is not expressed in the X-zone of postnatal wild-type mice, arguing that the effect of *Gata6* ablation on X-zone development is either a non-cell autonomous phenomenon or that it occurs in fetal adrenal cells that co-express *Gata6* and *Sf1*-cre (106). Recently, Sergei Tevosian’s laboratory reported that *Gata4*/*Gata6* double knockout mice generated with *Sf1*-cre exhibit severe adrenal hypoplasia; female double knockout mice die from adrenocortical insufficiency, whereas their male counterparts survive due to heterotopic glucocorticoid production by cells in the testes (107).

Circumstantial evidence from other organ systems suggests that GATA6 may modulate developmental signaling pathways in the adrenal cortex. In epithelial cells of the lung and intestine, GATA6 interacts with the WNT/β-catenin and TGF-β signaling pathways to regulate the balance between stem/progenitor cell expansion and differentiation (108–113). Hindlimb buds express *Gata6* in an anterior-posterior gradient, and conditional deletion of *Gata6* in limb bud mesenchyme of mice leads to ectopic expression of *Shh* and its target gene *Gli1*. The mutant mice develop hindlimb preaxial polydactyly. Conversely, enforced expression of *Gata6* in the limb bud represses expression of Shh and results in hypomorphic limbs. In an analogous fashion, GATA6 may repress transcription of *Shh* and *Gli1* in the adrenal cortex. Consistent with this notion, *Gli1* has been shown to be upregulated in the adrenal glands of gonadectomized *Gata6^flox/flox^;Sf1*-cre mice (106).

## Summary and Perspectives

The continual remodeling of the zones of the adrenal cortex requires the precise control of cell growth and differentiation. The process involves distinct pools of stem/progenitor cells in the capsule, subcapsule, and elsewhere. Direct lineage conversion of mature steroidogenic cells is also integral to adrenocortical zonation and remodeling. The pathways involved are complex and redundant so as to fulfill the offsetting goals of organ homeostasis and stress adaptation. Disruption of these pathways can lead to neoplasia.

Although much has been learned about the regulation of adrenocortical homeostasis and regeneration, there are still many unanswered questions. It has proven difficult to isolate and characterize adrenocortical stem cell populations, and we do not know how these populations vary with age. Nor do we understand the relative contributions of the hedgehog, DLK1, FGF, and WNT/β-catenin signaling pathways to adrenocortical differentiation, or how these pathways interface with classic endocrine signaling systems, such as the RAAS and the HPA axis. The positional cues that mediate differentiation during centripetal (or centrifugal) migration also remain enigmatic. In other epithelial organs (e.g., liver, intestine, and lung) the development of *in vitro* systems, such as organoid cultures and induced pluripotent stem cell models, has helped to elucidate the regulation of differentiation (114). To date, there has been little progress in the development of *in vitro* models to study adrenocortical differentiation. Hopefully, such techniques will emerge in the coming years and help drive the field forward.

## Conflict of Interest Statement

The authors declare that the research was conducted in the absence of any commercial or financial relationships that could be construed as a potential conflict of interest.
